# Cerebro-Cardiovascular Risk, Target Organ Damage, and Treatment Outcomes in Primary Aldosteronism

**DOI:** 10.3389/fcvm.2021.798364

**Published:** 2022-02-02

**Authors:** Xiao Lin, Muhammad Hasnain Ehsan Ullah, Xiong Wu, Feng Xu, Su-Kang Shan, Li-Min Lei, Ling-Qing Yuan, Jun Liu

**Affiliations:** ^1^Department of Radiology, The Second Xiangya Hospital, Central South University, Changsha, China; ^2^Department of Endocrinology and Metabolism, National Clinical Research Center for Metabolic Diseases, The Second Xiangya Hospital, Central South University, Changsha, China; ^3^Clinical Research Center for Medical Imaging in Hunan Province, Changsha, China; ^4^Department of Radiology Quality Control Center in Hunan Province, Changsha, China

**Keywords:** primary aldosteronism, aldosterone, cerebro-cardiovascular risk, target organ damage, mechanism, treatment

## Abstract

Primary aldosteronism (PA) is the most common type of endocrine hypertension, and numerous experimental and clinical evidence have verified that prolonged exposure to excess aldosterone is responsible for an increased risk of cerebro-cardiovascular events and target organ damage (TOD) in patients with PA. Therefore, focusing on restoring the toxic effects of excess aldosterone on the target organs is very important to reduce cerebro-cardiovascular events. Current evidence convincingly demonstrates that both surgical and medical treatment strategies would benefit cerebro-cardiovascular outcomes and mortality in the long term. Understanding cerebro-cardiovascular risk in PA would help clinical doctors to achieve both early diagnosis and treatment. Therefore, in this review, we will summarize the cerebro-cardiovascular risk in PA, focusing on the TOD of aldosterone, including brain, heart, vascular system, renal, adipose tissues, diabetes, and obstructive sleep apnea (OSA). Furthermore, the various treatment outcomes of adrenalectomy and medical treatment for patients with PA will also be discussed. We hope this knowledge will help improve cerebro-cardiovascular prognosis and reduce the incidence and mortality of cerebro-cardiovascular events in patients with PA.

## Introduction

Primary aldosteronism (PA) is a clinical syndrome mainly characterized by hypertension, suppressed levels of plasma renin, and autonomous plasma aldosterone overproduction. Recognition of the prevalence of PA has increased from <1% to over 10% of patients with hypertension, with the in-depth knowledge and application of the screening-confirmation-typing system (1). Moreover, the prevalence of PA in patients with refractory hypertension ranges from 8.9 to 33% (2). Therefore, PA is the most common type of endocrine hypertension. However, PA is often underestimated, and patients with untreated (or inappropriately treated) PA have an increased risk of cardiovascular events and target organ damage (TOD). A substantial body of clinical studies have demonstrated that undiagnosed PA is associated with stroke, heart failure (HF), diabetes mellitus (DM), obstructive sleep apnea (OSA), renal failure, and other consequential cardiovascular events, along with poorer health-related quality of life (QoL), even premature death (3–8). Prolonged exposure to excess aldosterone has a toxic effect on target organs, including the brain, heart, vascular system, kidney, adipose tissues, and OSA, which could increase the incidence and mortality of cerebro-cardiovascular events in patients with PA (4, 5). Most importantly, the rate of TOD, to a large extent, could be reversed *via* removing the toxic effects of excess aldosterone with either adrenalectomy (ADX) or treatment with a mineral ocorticoid receptor antagonist (MRA) (9). Therefore, this review aims to discuss the new evidence linking aldosterone to the TOD and treatment outcome of PA, thereby hoping to help decrease the risk of developing cerebro-cardiovascular and renal complications in patients with PA.

## Mechanisms of Aldosterone on Target Organs

Aldosterone, a mineral corticoid hormone, is synthesized in the zona glomerulosa of the adrenal glands. Aldosterone is one of the effector molecules of the renin-angiotensin-aldosterone system (RAAS), whose synthesis and secretion are stimulated by angiotensin II (Ang-II) through the angiotensin I receptor (ATI-R) in the adrenal cortex (10). The genomic effects of aldosterone occur through binding to the mineral ocorticoid receptor (MR), translocating to the nucleus, interacting with DNA, and thus promoting the transcription of genes that regulate the transport of sodium and potassium and fluid balance (11). Aldosterone induces rapid cellular responses by modulating intracellular calcium (${\text{Ca}}_{2}^{+}$) and cyclic adenosine monophosphate (cAMP) levels; sodium/hydrogen (Na^+^/H^+^) exchanger activity; and phosphorylation of signaling molecules, including protein kinase C (PKC), epidermal growth factor receptor (EGFR), mitogen-activated protein kinases (MAPKs), (including c-Jun NH2-terminal kinase), and extracellular signal-regulated kinases (ERKs) 1/2 (12–14). Recent insights into sodium and potassium have demonstrated that excess aldosterone promotes epithelial sodium channel (ENaC) activity and facilitates renal outer medullary K^+^ channel (ROMK) activity to increase the sodium chloride cotransporter (NCC) and pendrinapical abundance in the late but not in the early distal convoluted tubule, which allows sodium/potassium exchange through the ENaC, ROMK, and big potassium channel (15). Wu et al. demonstrated that oral co-administration of fludrocortisone acetate (a potent mineral ocorticoid) and KCl in patients with PA is associated with reduced pendrin and enhanced ROMK in urinary extracellular vesicles (16). Based on this, Stavropoulos et al. assumed that pendrin inhibition might confer an efficacious therapeutic option for patients with PA (17).

**Figure 1:**
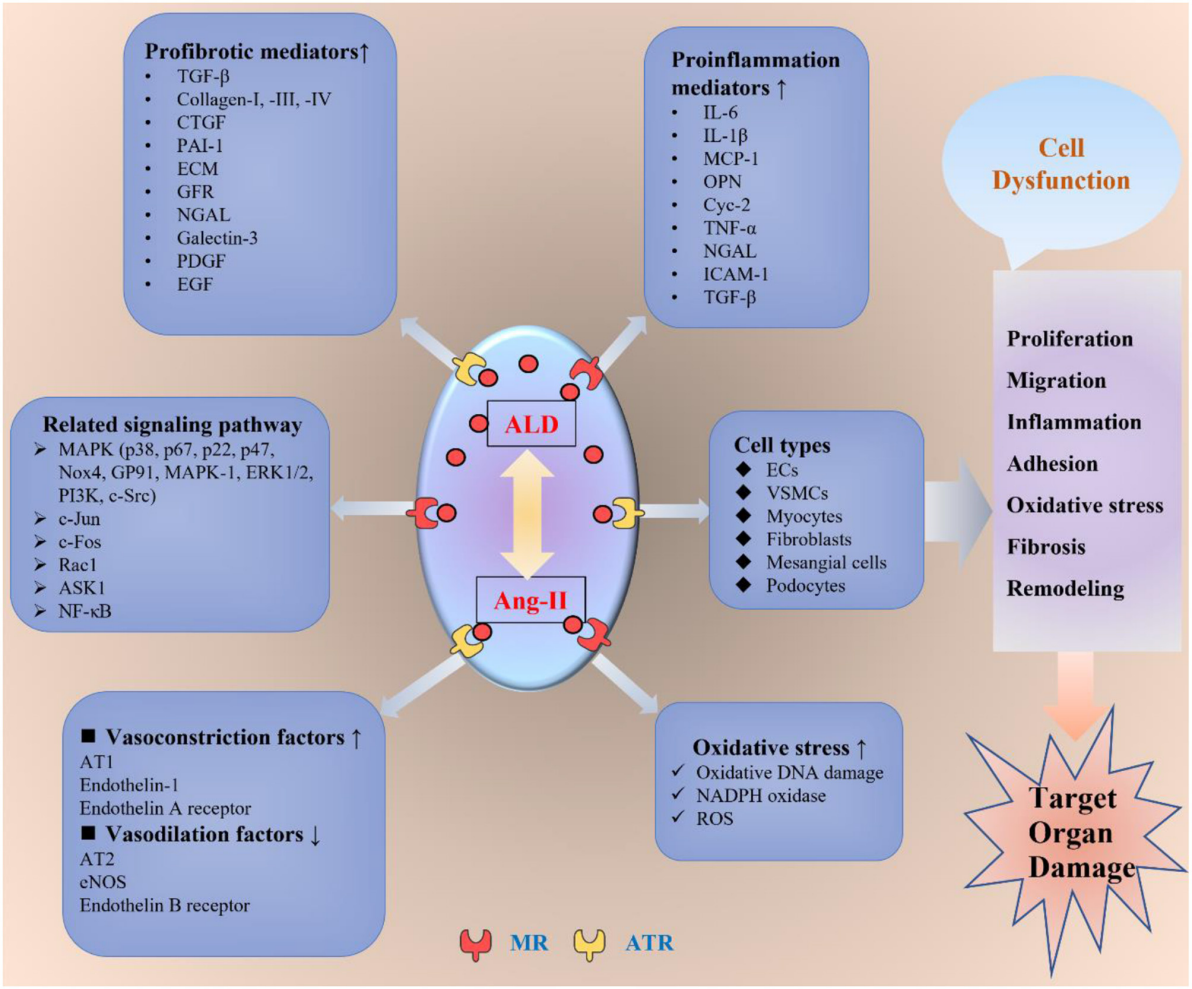
The non-genomic mechanisms of aldosterone-modulating TOD. ALD interacts with Ang-II by binding to the MR or ATR to exert its toxic effect on the target organs. ALD promotes inflammation, oxidative stress, fibrosis, migration, proliferation, adhesion, endothelial dysfunction, and vascular remodeling through different kinds of mediators and signaling pathways in various cells, including ECs, VSMCs, myocytes, fibroblasts, mesangial cells, and podocytes. ALD, aldosterone; Ang-II, angiotensin II; ATR, angiotensin receptor; MR, mineral ocorticoid receptor; AT1, angiotensin receptor 1; AT2, angiotensin receptor 2; ECs, endothelial cells; VSMCs, vascular smooth muscle cells; eNOS, endothelial nitric oxide synthase; TGF-β, transforming growth factor β; CTGF, connective tissue growth factor; PAI-1, plasminogen activator inhibitor 1; ECM, extracellular matrix; GFR, growth factor receptor; NGAL, neutrophil gelatinase-associated lipocalin; PDGF, platelet-derived growth factor; EGF, epidermal growth factor; IL-1β, interleukin 1β; IL-6, interleukin 6; MCP-1, monocyte chemoattractant protein-1; OPN, osteopontin; TNF-α, tumor necrosis factor-α; ICAM-1, intercellular adhesion molecule-1; NADPH, nicotinamide adenine dinucleotide phosphate; ROS, reactive oxygen species; ASK1, apoptosis signal-regulated kinase 1; ERK1/2, extracellular signal-regulated kinase 1/2; PI3K, phosphoinositide 3-kinase; NF-κB, NF-kappaB; TOD, target organ damage.

Besides, the aldosterone non-genomic pathways are through the AT1-R, G-protein-coupled receptor, and EGFR (18). These receptors include the MAPK/ERK1/2/p38 signaling pathways, mediating vascular remodeling, inflammation, hemodynamic alterations, nephrosclerosis, and fibrosis (19, 20), as well as being involved in cardiovascular, renal, and metabolic diseases (21, 22). Therefore, the outcome of aldosterone excess would induce structural and functional alterations in the heart, kidney, and vascular system, which leads to the development of cardiac hypertrophy, stroke, coronary heart disease, nephrosclerosis, vascular inflammation, sclerosis, myocardial infarction, fibrosis, and tissue remodeling (5, 23–25). The major pathophysiological non-genomic mechanisms involved in the impact of aldosterone on target organs are summarized in Figure 1, and part of the detailed mechanisms are discussed as follows.

### Aldosterone and Oxidative Stress

Multiple animal (rats and mice) studies have demonstrated that aldosterone and Ang-II potentiate each other's action in inducing oxidative stress *via* elevating levels of oxidative stress markers (malondialdehyde, procollagen type 1 amino-terminal propeptide et al.), leading to detrimental consequences in the target organs of patients with PA (26–28). Human red blood cells (RBCs) are anucleated cells and are particularly sensitive to oxidative assault. Bordin et al. verified that RBCs from patients with PA displayed membrane alterations and increased senescence *in vitro*, which were accompanied by increased high molecular weight aggregates and diamide-induced band 3 Tyr-P levels (29). Moreover, in an *in vivo* study, they demonstrated that, due to the MR-mediated response involving ligand specificity, aldosterone would induce MR activation and lead to RBC membrane alterations and IgG binding in patients with PA (30).

Aldosterone has been reported to activate nicotinamide adenine dinucleotide phosphate (NADPH) oxidase and increase reactive oxygen species (ROS) levels in several kinds of cells, including myocardial cells, endothelial cells (ECs), vascular smooth muscle cells (VSMCs), and mesangial cells in rats (31, 32). Petramala et al. firstly demonstrated increased oxidative stress characterized by increased serum levels of NADPH oxidase (Nox-2-derived peptide) and urinary excretion of isoprostanes in patients with PA (33). Besides, aldosterone would interact with Ang-II to activate NADPH oxidase through the phosphorylation and activation of p47phox and Rac1 (27, 28, 32), as well as c-Src-dependent mechanisms, in mice (26). Aldosterone increased rat myocyte ROS production by the non-genomic activation of NADPH oxidase, which in turn triggers apoptosis of myocyte associated with the activation of apoptosis signal-regulating kinase 1 (ASK1) (34). Besides, aldosterone activating the MR in ECs and VSMCs, derived from mice, rats, or humans, is associated with ROS production through increasing the expression and activity of NADPH oxidases in the heart (26, 35–37). Aldosterone decreases the expression of glucose-6-phosphate dehydrogenase (G6PD), shifting the balance toward increased oxidative stress in bovine and human ECs (38). In human pulmonary artery ECs, aldosterone increases ROS, which in turn modifies the cysteinyl thiols in the endothelial nitric oxide synthase (eNOS) activating region of the endothelin B receptor, thus decreasing endothelin-1-stimulated eNOS activity (37). In addition, MRs in SMCs contribute to Ang-II-induced vascular oxidative stress both in mice and humans (39). Moreover, oxidative stress, in turn, promotes rat SMC senescence (40). Another important signaling molecule in VSMCs, c-Src, is a critical proximal regulator of NAD(P)H oxidase (41), and aldosterone rapidly increases activation of MAPKs (p38MAPK, c-Jun NH2-terminal kinase, and ERK1/2) through c-Src-dependent pathways in mice (26). In addition, chronic treatment with aldosterone will induce MAPK activation *via* a ROS-dependent pathway and then, increase the expression of p67phox, p22phox, Nox4, Gp91phox, p47phox, and Rac1 in the rat kidney (42–44), and it also induces apoptosis in rat mesangial cells (45).

### Aldosterone and Inflammation

Early study has demonstrated that rats treated with aldosterone have perivascular leucocyte infiltration and increased expression of interleukin (IL)6, IL-1β, osteopontin (OPN), monocyte chemoattractant protein 1 (MCP-1), and cyc-2 in the kidney (46). Besides, aldosterone participated in the process of inflammation in mice by increasing the expression of adhesion molecule-1 and the activation of c-Jun and c-Fos in response to pro-inflammatory stimuli (47). Eissler et al. also demonstrated that aldosterone causes the overexpression of toll-like receptor4 (TLR4) and the higher expression of inflammatory cytokines (TNF-α, IL-1, and MCP-1) in rat cardiac tissue (48). Moreover, aldosterone had pro-inflammatory effects by increasing neutrophil gelatinase-associated lipocalin (NGAL) expression in mice dendritic cells, macrophages, and peripheral blood mononuclear cells (49). In mice dendritic cells, aldosterone also induces the secretion of the pro-inflammatory cytokines IL-6 and transforming growth factor β (TGF-β) *via* MR activation (50).

### Aldosterone and Fibrosis

In experiments with rats and mice, aldosterone administration has been associated with an increase in TGF-β, connective tissue growth factor (CTGF), and collagen gene expression that is accompanied by kidney fibrosis (51–54). Aldosterone has been reported to increase the production of plasminogen activator inhibitor-1 (PAI-1) and subsequent extracellular matrix (ECM) accumulation in the development of glomerulo sclerosis and SMC stiffness (55–58). Besides, aldosterone has been reported to stimulate collagen gene expression and synthesis in cultured fibroblasts *via* activation of cellular ERK1/2 phosphorylation (59) and increase the mRNA levels of collagens I, III, and IV in rat glomerular mesangial cells (60). Moreover, aldosterone stimulates fibronectin synthesis through MR-dependent activation and phosphorylation of the c-Jun N-terminal kinase in rats (61). Aldosterone also induces expression of OPN in rat fibroblasts (62) and stimulates rat fibroblasts, resulting in rapid activation of growth-factor receptors (GFRs) and the induction of phosphoinositide 3-kinase/mitogen-activated protein kinase (PI3K/MAPK) signaling, which stimulates proliferation of fibroblasts (63). In addition, in human studies, NGAL has been shown to increase the production of galectin-3 and collagen I through the NF-κB pathway (64, 65) and acted through the TGF-β signaling, epidermal growth factor (EGF), platelet-derived growth factor (PDGF), and their receptors, thus contributing to the development of fibrosis (66, 67).

## Aldosterone and TOD

**Figure 2:**
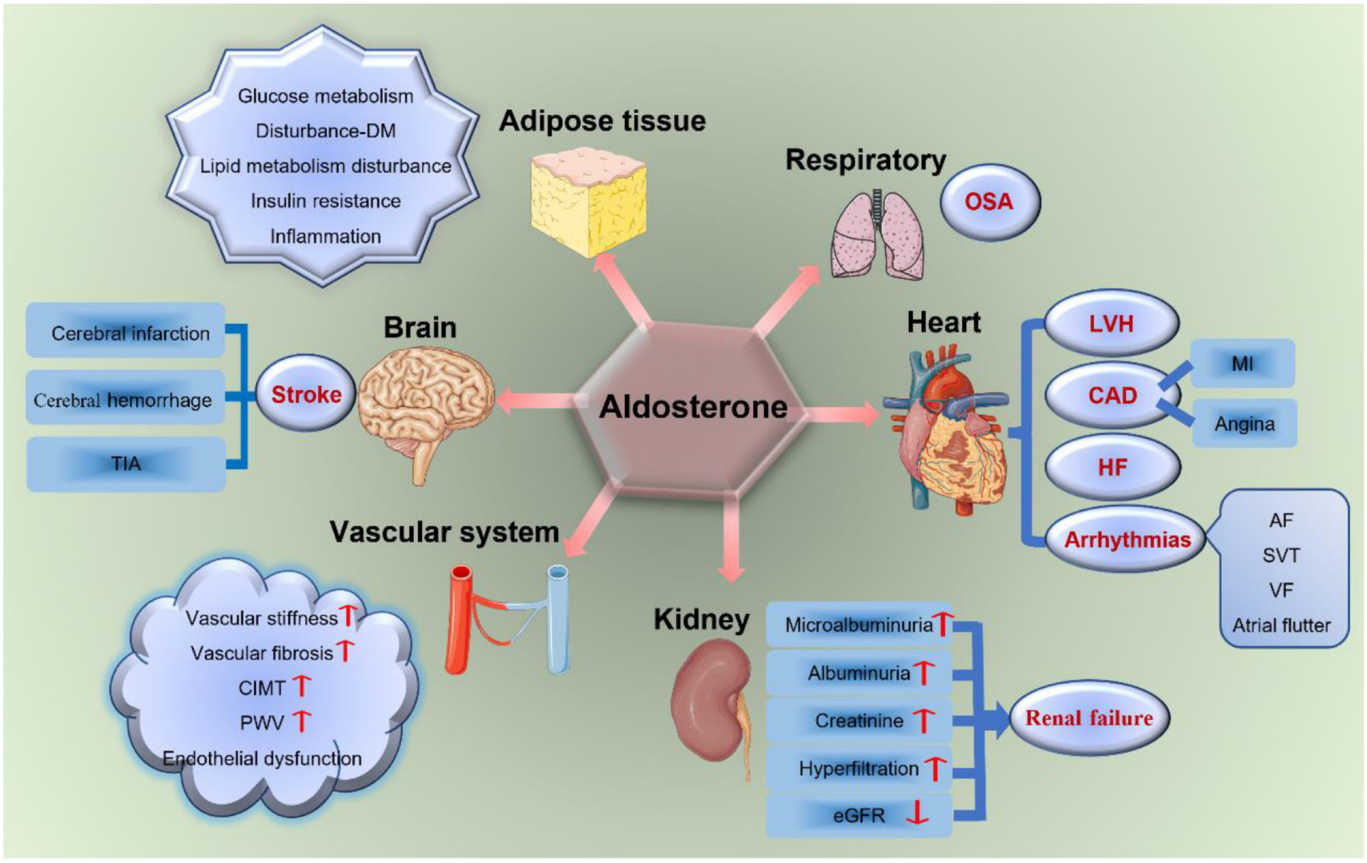
Aldosterone and TOD. Aldosterone has a toxic effect on target organs, including the brain, heart, vascular system, kidney, respiratory system, and adipose tissues. It increases the incidence of stroke, LVH, CAD, HF, arrhythmias, renal failure, vascular fibrosis and stiffness, endothelial dysfunction, glucose and lipid metabolism disturbances, insulin resistance, OSA, and so on. TIA, transient ischemic attack; DM, diabetes mellitus; LVH, left ventricular hypertrophy; CAD, coronary artery disease; MI, myocardial infarction; HF, heart failure; AF, atrial fibrillation; SVT, sustained ventricular tachycardia; VF, ventricular fibrillation; eGFR, estimated glomerular filtration rate; CIMT, carotid intima-media thickness; PWV, pulse wave velocity; OSA, obstructive sleep apnea; TOD, target organ damage.

With this new evidence, excessive aldosterone is regarded as an important determinant of the cerebro-cardiovascular risk profile in patients with PA, which has a toxic effect on the cerebro-cardiovascular system. Moreover, aldosterone is associated with severe TOD (including the brain, heart, kidney, vascular system, adipose tissues, and OSA) as shown in Figure 2, independently from blood pressure (BP) levels (7, 25).

### Toxic Effects of Aldosterone on Stroke

Stroke is defined as ischemic or hemorrhagic cerebrovascular disease, including cerebral infarction, cerebral hemorrhage, or transient ischemic attack (TIA) (9, 68). Experimental and human studies have verified that excess aldosterone promotes cerebral vascular oxidative stress, inflammation, and endothelial dysfunction, which increases the risk of stroke, independent of BP and other risk factors (9, 43, 68, 69). Cerebrovascular accidents or TIAs are more frequent in patients with PA than essential hypertension (EH), and cerebral infarction is the most frequent of the cerebrovascular events (70).

In animal studies, as early as 1992, Kim et al. found that plasma aldosterone was increased in a 25-week-old stroke-prone spontaneously hypertensive rat (SHRSP) (71). Accordingly, Enea et al. reported that plasma aldosterone levels were significantly increased and enhanced stroke development in SHRSP rats (from 442 ± 56.5 pg/ml to 739 ± 125.7 pg/ml) vs. Wistar-Kyoto rats (72, 73). Other studies have reported that rats fed aldosterone chronically experienced strokes, and treatment with a MRA ameliorated the effects (43, 74). A mechanism study demonstrated that Nox2-containing NADPH oxidase-mediated aldosterone induced increases in ROS production and endothelial dysfunction in cerebral arteries from mice, independently of BP changes (75).

In clinical studies, as early as 1998, Litchfield et al. found that patients with glucocorticoid-remediable aldosteronism tended to have an early onset of stroke (cerebral hemorrhage), which was related to increased aldosterone levels (68). Accordingly, Cristiana et al. verified that prolonged exposure to elevated aldosterone levels could increase the incidence of stroke or TIA in patients with PA when compared to patients with EH (9). Moreover, Takeda et al. investigated the incidence of cerebrovascular complications in 224 cases of PA in Japan and found that there were 14 cases of cerebral hemorrhage and 10 cases of cerebral infarction (76). Another retrospective study showed that stroke and TIA were increased significantly in patients with PA compared to those with EH (10.4 vs. 4.9%) (77), and the latest multicenter study also demonstrated that the prevalence of stroke was about 7.4% in Japan (78). In addition, Milliez et al. reported 12.9% of patients with PA had a history of stroke (4). However, Miyaji et al. discovered the incidence of PA in patients with acute stroke was just 4.% (79). The reasons to underestimate this incidence of stroke in patients with PA might be due to the demographic bias and non-standard confirmatory test strategies.

### Toxic Effects of Aldosterone on the Heart

Structural and functional abnormalities of the heart are common consequences of hypertensive states (80), and about 14 to 35% of patients with PA have cardiovascular complications, including myocardial hypertrophy, myocardial fibrosis, coronary artery disease (CAD), heart failure (HF), and atrial fibrillation (AF) (7, 81–83). Moreover, several large studies from different parts of the world have demonstrated that among patients with hypertension of equal severity and duration, those with PA have significantly more cardiovascular pathology than those with essential hypertension (5, 84, 85). Experimental and clinical studies have also demonstrated that both cardiomyocytes and cardiac fibroblasts express MR with a high affinity for aldosterone, and they could be activated by high aldosterone levels (86, 87). Based on that, aldosterone has been proved to affect both the structure and function of the heart and is regarded as a potential cardiovascular risk factor in patients with PA (86–88). The role of aldosterone in left ventricular hypertrophy (LVH), CAD, HF, and arrhythmias will be discussed as follows.

#### Aldosterone and LVH

Left ventricular (LV) remodeling is a process of changes in LV size, shape, texture, and function regulated by various stimuli including aldosterone, and it includes two parts in PA: LVH and fibrosis (89). PA is associated with a higher degree of LVH and increased LV mass index (LVMI) when compared to matched EH, and LVH leads to changes in cardiac size, mass, geometry, and function (83). LVH is the most common cardiac structural abnormality induced by excess aldosterone, independently of its effects on BP (7, 74, 90, 91). Myocardial hypertrophy evaluated by electrocardiogram or echocardiography, especially LVH, was reported to be about two times as frequent in patients with PA when compared to that in otherwise similar patients with EH (4, 83, 92). The latest study demonstrated that autonomous aldosterone secretion levels, not the basal aldosterone concentration itself, were correlated significantly with LVMI, even after adjusting for age and BP (88). A recent meta-analysis by Monticone et al. (5) based on a pooled population from 20 studies, totaling 5,672 patients, documented that LV mass was higher in patients with PA compared to that in counterparts with EH (mean difference ranging from 20 to 69 g/m^2^ for LV mass/BSA and from 5 to 58 g/m^2.7^ for LV mass/h^2.7^).

In animal studies, the experimental study suggested that long-term aldosterone-salt treatment in rats displayed an increase in cardiac fibrosis and LVH (93). The endogenous aldosterone produced in the heart directly stimulates the hypertrophy of ventricular myocytes in neonatal rats *via* activation of ERK, JNK, and protein kinase C-α (PKC-α), affecting cardiac hypertrophy and function in hypertensive rats (94, 95). Besides, aldosterone has been shown to increase the size of rat cardiac myocytes, along with an increase in ROS and nitric oxide (NO) (96). Moreover, elevated aldosterone has been shown to promote myocardial hypertrophy and fibrosis in the rat model of aldosterone system overexpression (97).

In clinical studies, several cross-sectional studies have reported both LV wall thickness and LV dimension were increased in patients with PA (98–101). The mean interventricular septum and posterior wall thicknesses were increased, leading to higher rates of LVH in patients with PA relative to individuals with EH (101). The other two types of research have demonstrated that patients with PA had reduced diastolic function and increased LV wall thickness, even in the absence of arterial hypertension (102, 103). Exclusive analysis of a prospective study confirmed the association of PA with higher LV internal dimensions and higher LVMI compared with those in subjects with EH (104). Besides, the prevalence of LVH had no significant gender difference in patients with PA (105). What is more, Chen et al. demonstrated that patients with PA showed not only LV abnormalities but also impaired right ventricular function because of hyper aldosteronism (106).

#### Aldosterone and CAD

Coronary artery disease (CAD) often includes myocardial infarction (MI) and stenocardia, and MI is the most common manifestation of CAD. Mihailidou et al. demonstrated that aldosterone increased the incidence of MI, aggravating cardiac damage (107). Increasing production of cardiac aldosterone has been shown to increase the risk of MI, and in turn, MI could raise aldosterone synthase mRNA (the terminal enzyme of aldosterone synthesis) and the level of aldosterone in rats (108). Aldosterone has also been shown to induce a vascular inflammatory phenotype in rat heart and strongly increased cyclooxygenase 2 (COX-2) in ventricular cardiomyocytes after MI (109). Besides, increased levels of aldosterone have been shown to activate MR in the brain and then enhance apoptosis both in rat myocytes and nonmyocytes in the peri-infarct and infarct areas post-MI, contributing to the inflammatory response (110).

Patients with PA experienced MI or unstable angina, requiring angioplasty more frequently than those with EH, both at PA diagnosis, during follow-up, and in the overall period of the study (4, 9, 77, 111). Milliez et al. demonstrated non-fatal MI was diagnosed in 4.0% of 124 patients with PA and 0.6% of 456 patients with EH (4). Subsequently, Catena et al. demonstrated an increased risk of MI in patients with PA at diagnosis and during a 7-year follow-up study (9). In addition, a multicenter study in Japan involving 2,582 patients with PA found that the prevalence of CAD (including MI or angina) was 9.4% (78).

#### Aldosterone and HF

Heart failure (HF) is defined as, “a complex clinical syndrome that could result from any structural or functional cardiac disorder, which impairs the ability of the ventricle to fill or eject blood” (77, 111, 112). HF has been singled out as a clinical and public health problem and is associated with significant morbidity, mortality, and healthcare expenditures (112). The risks of developing HF requiring hospitalization and mortality were significantly higher in patients with PA than EH controls (111). Although Takeda et al. showed the incidence of congestive heart failure (CHF) in the PA group was a little lower than that in the EH group in 1995, this difference did not reach statistical significance (76). However, a large cohort study confirmed that the incidence of HF was much higher in patients with PA compared to that in patients with EH (111). Moreover, a multicenter study in Japan also found that the prevalence of HF (about 0.6%) was higher among patients with PA compared to that among EH controls (78).

#### Aldosterone and Arrhythmias

Atrial fibrillation (AF) is one of the most important and prevalent types of arrhythmias and imposes an increasing burden on the healthcare system, owing to the need for lifelong care and pharmacological treatment, which is associated with an increased risk of cardiovascular events (113). In patients with PA, aldosterone not only increased cardiac structural remodeling (such as atrial fibroblasts, LVH, and interstitial collagen) (114) but also promoted AF by altering repolarizing potassium currents, leading to action potential shortening in rats (115). Furthermore, Lu et al. reported that aldosterone enhanced the expression of Kv1.5, a promising target for the treatment of AF, by activating ROS-dependent phosphorylation of Smad 2/3 and ERK 1/2 in a rat AF model (116).

A growing body of evidence has demonstrated increased sustained arrhythmias, including AF, sustained ventricular tachycardia, atrial flutter, and ventricular fibrillation (VF), in patients with PA compared to those with EH (77, 111, 117, 118). Milliez et al. reported a history of AF in 7.3% of patients with PA (4), which was consistent with the latest study that showed a marked increase in the relative risk of AF (12.1-fold) in patients with PA compared to patients with EH (119). In addition, another study also reported that patients with PA had a 7.2-fold higher prevalence of history or current AF than patients with EH (101). Consistently, both a multicenter study and a large cohort study confirmed that the incidences of arrhythmias, especially AF, were all significantly higher among patients with PA compared to those among other hypertensive patients (78, 120). Besides, Catena et al. demonstrated an increased risk of sustained arrhythmias in patients with PA at diagnosis compared to patients with EH during a 7-year follow-up study (9). More seriously, PA with hypokalemia has been shown to increase the risk of life-threatening ventricular arrhythmias (121).

### Toxic Effects of Aldosterone on the Vascular System

Aldosterone has been shown to regulate vascular contractility, cell growth, and apoptosis. Aldosterone-mediated EC growth and endothelium-mediated regulation of vasore activity *via* activation of G protein-coupled estrogen receptor (GPER) were observed in rats (122). Aldosterone has been shown to interact with Ang-II to increase apoptosis of rat microvascular ECs *via* upregulation of protein tyrosine phosphatase 1B (PTP1B) expression and inhibition of the PI3K/Akt pathway (123). In addition, in an *in vitro* study, aldosterone was shown to contribute to an increase of collagen synthesis and fibrosis in mice VSMCs (8, 26).

It is well documented that excess aldosterone is responsible for increased arterial wall stiffness due to the morphological and functional abnormalities of the blood vessel wall (3, 6, 8). The mechanisms leading to structural abnormalities include increases in both arterial wall stiffness (6) and carotid intima-media thickness (CIMT) (124), as well as abnormal endothelial function (8). The excess aldosterone generates oxidative stress to cause endothelial dysfunction and collagen remodeling or reduce the bioavailability of NO to directly affect EGFR, both of which lead to an increase in fibrosis and vascular stiffness (125, 126). Furthermore, increased CIMT was more frequent in patients with PA than in controls with EH (8, 127, 128). In addition, pulse wave velocity (PWV) is regarded to be another reliable marker of atherosclerosis and arterial stiffness. Several studies have demonstrated that PWV values, as well as brachial-ankle/heart-ankle PWV, increased in patients with PA compared to those in controls with EH, and this difference was independent of BP levels (125).

What is more, excess aldosterone has been shown to cause reduced endothelial progenitor cell (EPC) vascular elasticity, proliferation, differentiation, and migration in patients with PA (129), and the deficiency of EPCs may result in increased aortic stiffness and vascular damage (130). Moreover, endothelial inflammation contributed to the negative remodeling of the cerebral vasculature, which made the vessel wall less flexible and further impaired dilatation of the cerebral vessels during the stroke (131). Furthermore, pronounced fibrosis of small resistance arteries was also detected in patients with PA compared with BP-matched patients with EH (132).

### Toxic Effects of Aldosterone on the Kidney

Clinical studies have indicated that plasma aldosterone concentration (PAC) was associated with renal dysfunction, and the incidence of renal complications was higher among patients with PA when compared to patients with EH at equivalent BPs (7, 133). A high PAC could affect more pronounced renal damage, including renal glomeruli and tubules (134–136). The latest histopathological analysis of patients with PA demonstrated that both mineral ocorticoid receptor and 11β-hydroxysteroid dehydrogenase type 2 were significantly higher in the renal tubules of patients with hyperaldosteronism, which resulted in interstitial fibrosis and segmental glomerulosclerosis. In the meantime, in those with hyperaldosteronism, glomerular size was significantly larger, luminal stenosis tended to be more marked, and arteriolar hyalinization was significantly more pronounced, but the intima-to-media ratio was significantly lower (134). In addition, the effective markers of kidney damage, including β2-microglobulin (β2-MG), urinary liver fatty acid-binding protein (L-FABP), and N-acetyl-β-D-glucosaminidase (NAG) for tubular damage, as well as urinary albumin-creatinine ratio (ACR) for glomerular damage, were significantly correlated with the PAC (136). In addition, severe vascular and glomerular sclerosis, fibrinoid necrosis and thrombosis, interstitial leukocyte infiltration, and tubular damage and regeneration were observed in aldosterone-treated rats, which leads to renal injury and fibrosis (46).

The structural renal damage induced by a high PAC in patients with PA may be associated with unfortunate outcomes, such as renal injury and renal failure (137, 138). Patients with PA presented more frequently with microalbuminuria and albuminuria, lower eGFR, and increased creatinine levels (111, 136, 139, 140). Studies have reported that albuminuria and renal failure were found in a range from 8 to 24% in subjects with PA (138, 141). Also, 24-h microalbuminuria was significantly greater in patients with PA compared to controls with EH in the PAPY study (142). Both Reincke et al. and Kawashima et al. found the prevalence of albuminuria was increased, and eGFR was lowered greatly in patients with PA, independent of other known risk factors (143, 144). Moreover, a recent meta-analysis study including 6,056 patients with PA also verified that patients with PA had an increased eGFR compared with other hypertensive patients [by 3.37 ml/min IQR (0.82–5.93)] and more severe albuminuria [standard mean difference.55 (0.19–0.91)], resulting in an association with microalbuminuria [odds ratio (OR): 2.09 (1.40; 3.12)] and proteinuria [OR: 2.68 (1.89; 3.79)] (145). The follow-up study further demonstrated that the greater risk of impairment of kidney function in patients with PA was independent of BP degree (146).

Another crucial influence of aldosterone excess in patients with PA is relative hyperfiltration (147). Most patients with long-standing PA have some degree of renal insufficiency, but aldosterone excess-induced glomerular hyperfiltration may mask mild to moderate underlying renal failure (148). The early sign of long-lasting hyperfiltration is microalbuminuria (149), and preventing the development of microalbuminuria by recognizing glomerular hyperfiltration becomes an important target in the management of PA (150). Therefore, the relative hyperfiltration beyond the effect of hypertension in PA could mislead clinicians as to the interpretation of normal and abnormal renal function by examining eGFR data only. Therefore, clinicians should be aware of possible underlying renal damage in patients with PA even when the eGFR is normal.

### Toxic Effects of Aldosterone on Adipose Tissue

Adipose tissue is a complex, essential, and highly active metabolic and endocrine organ (151). It has been believed to have autocrine, paracrine, and endocrine functions and plays a key role in the pathogenesis of glucose and lipid metabolism disturbances and insulin resistance (IR) in patients with PA (152, 153). A high PAC may induce adipose tissue dysfunction and lead to inflammation, fibrosis, and a high incidence of metabolic syndrome in patients with PA (154, 155). Years ago, Rondinone et al. demonstrated that MRs were presented in adipocytes isolated from rats and aldosterone could induce differentiation of 3T3-L1 cells because of the presence of specific mineral ocorticoid-binding sites (156).

Both subcutaneous adipose tissue (SAT) and visceral adipose tissue (VAT) were observed to have a potential interplay with aldosterone in patients with PA (157–159). Studies have reported that the number and affinity of insulin receptors in SAT were reduced significantly, which resulted in glucose intolerance and reduced insulin sensitivity in patients with PA (160, 161). Besides, the expression of PCK1, ADIPOQ, PLIN, and PPARG in VAT were inversely correlated with excess aldosterone levels, which in turn contribute to the IR observed in patients with PA (159). Mechanism studies have demonstrated that aldosterone-reduced glucose uptake in human adipocytes by reducing GLUT4 cell-surface localization and phosphorylation of IRS1, PI3K, and AKT (162), as well as insulin signaling, was impaired in a rat model of PA (163). In addition, aldosterone excess was also associated with a reduction of both leptin and adiponectin expression in VAT in patients with aldosterone-producing adenoma (APA) (164, 165). Besides, elevated aldosterone levels are associated with elevated circulating resistin levels and cardiac morphological changes, independent of the presence of metabolic syndrome in patients with PA (154). However, other studies have shown that the correlation between the PAC and the percentage of VAT was not evident in patients with APA (158), and patients with APA had smaller visceral fat areas than their counterparts with EH (166).

### Toxic Effects of Aldosterone on Diabetes Mellitus (DM)

Primary aldosteronism (PA) is frequently associated with impaired insulin sensitivity and an increased risk of developing DM. A multi-institutional, cross-sectional study demonstrated that DM was an independent risk factor in cardio-cerebrovascular events and renal complications in patients with PA (167). Elevated aldosterone levels were independently associated with IR, as observed by Kumagai et al. in a 10-year prospective study (168). Recent studies have further demonstrated that patients with APA presented with increased levels of not only aldosterone but also cortisol *via* the expression of both CYP11B1 and CYP11B2. CYP11B1 is required for the synthesis of cortisol and 11β-hydroxy and rostenedione, and CYP11B2 catalyzes aldosterone synthesis (169). Gerards et al. demonstrated that impaired glucose metabolism was associated with cortisol cosecretion, which increased the risk of type 2 DM in patients with PA (170). Besides, the coexistence of autonomous cortisol secretion was associated with poor clinical outcomes and psychological symptoms in patients with PA (171, 172). A recent prospective cohort study demonstrated that PA caused decreased insulin secretion and an increased rate of insulin clearance, which lead to glucose intolerance (173). Physically, insulin and glucose are delivered to different organs by the circulation, and they enter specific cells through NO-dependent transport (174). In patients with PA, excess aldosterone and MR activation increase ROS and promote endothelial remodeling, resulting in VSMC-related IR (175), skeletal muscle-related IR (176), and adipocyte-related IR (177). Furthermore, the relationship between PA and the increased prevalence of abnormal glucose metabolism was strengthened by two meta-analyses. Subjects with PA had a higher risk of developing DM (OR 1.33, 95% CI1.01–1.74) and a higher prevalence of DM compared to subjects with EH (OR 1.55, 95% CI 1.01–2.36, *p* = 0.04) (5). They also had an increased prevalence of impaired fasting glucose [31.2% (95% CI15.81–46.60%)], impaired glucose tolerance [26.19% (95% CI 15.17–37.21%)], and DM [15.22% (95% CI9.93–20.51%)] (178).

### Aldosterone and OSA

Obstructive Sleep Apnea (OSA) is the most frequent secondary condition associated with resistant hypertension, and the association between OSA and PA has been a matter of debate (179). Recently, several efforts have been devoted to investigating the bidirectional relationship between aldosterone levels and OSA (180, 181). A small sample size (53 patients) study showed that 34% of hypertensive patients with OSA had PA (180). Besides, a prospective study that enrolled 207 patients with OSA showed the frequency of PA in patients with moderate-to-severe OSA was up to 21.3%, suggesting that moderate-to-severe OSA predicted the presence of PA (182). However, the Hypertensives with Primary Aldosteronism and OSA (HYPNOS) study demonstrated that the prevalence of PA in patients with OSA was 8.9% (11.8% of Caucasian ethnicity and 5.9% of Chinese ethnicity) (181), which is challenging the current recommendation of the 2016 Endocrine Society guidelines that recommend screening for PA in all hypertensive patients with OSA (183). But the latest study supported that rigorous screening for PA is cost-saving due to the aversion of cardiovascular risk even if screening was conservatively presumed to identify only 3% of new PA cases (184). What is more, aldosterone levels contributed to the severity of OSA in patients with PA (185). Accordingly, the HYPNOS study found the prevalence of OSA was 67.6% in the overall cohort of patients with PA (64.4% of Caucasian ethnicity, and 70.0% of Chinese ethnicity, respectively) (181). Another retrospective study in the Japanese population also showed that 55% of patients with PA were diagnosed with OSA (135). Interestingly, both of the studies by Wolley and Gaddam verified that treatment with a MRA in patients with PA significantly reduced the number of those with OSA (186, 187). Collectively, early screening of patients with moderate-to-severe OSA for PA could reduce TOD. Most importantly, improving the awareness of the impact of hyper aldosteronemia on OSA in patients with PA can help to reduce morbidity and mortality in patients with moderate-to-severe OSA.

## Treatment Outcomes of PA

Targeted treatment with either adrenalectomy (ADX) and MRA are the two common, well-documented treatments to improve the outcomes of patients with PA. The current practice guidelines recommend ADX for lateralized aldosterone excess, whereas bilateral lesions are treated using a MRA (183, 188). When ADX was performed for lateralized aldosterone excess, the cure rate for hypertension (a composite of patients who are cured or experience a marked improvement) was up to 82% and that of the biochemical aspects of PA was close to 100%. Even when antihypertensive treatment cannot be withdrawn after ADX, the number or the doses of antihypertensive drugs can be markedly decreased, and/or resistant hypertension can be resolved (189). However, it should be noted that aldosterone levels, which are the occupation of aldosterone target-MR, are tightly regulated by the RAAS system, and treatment with a MRA cannot fully replicate physiological control. Interestingly, ADX was also associated with a considerable improvement in several indexes of QoL (190). For patients who are not candidates for surgery, treatment with anMRA is a reasonable alternative to ADX. However, it is difficult to assess response to therapy in PA due to a lack of standardized outcome measures. Although the majority of subjects benefited from both surgical and medical therapies in observational studies (120, 191, 192), the absence of unifying criteria for therapeutic response has limited comparative investigations. In 2017, the Primary Aldosteronism Surgical Outcome (PASO) study published explicit criteria for biochemical and clinical outcomes after ADX, but the complexity of the PASO criteria significantly limits their utility in routine clinical settings. Because the PASO criteria do not reflect the secondary outcomes associated with PA, including cardiovascular and renal dysfunction, which are arguable of significant clinical importance (193).

Recent investigations of therapeutic outcomes in PA have evaluated alternative endpoints, including target organ function, QoL, and overall survival (194). Although overall mortality after the diagnosis of PA and initiation of specific treatment is similar to that of matched controls with EH, available evidence, based on predominantly retrospective and a few prospective observational studies, shows that treatment of PA by ADX or treatment with anMRA reduced the all-cause morbidity of untreated PA, thereby reducing overall mortality (9, 195). Besides, the TOD induced by excess aldosterone could also be reversed, to a great degree, *via* ADX or treatment with anMRA (196, 197). Several recent studies (2011–2021) evaluating ADX vs. MRA therapy are discussed below and summarized in Table 1.

**Table 1:** A summary of selected recent studies (2011–2021) comparing medical and surgical treatments of PA.

**Study**	**Source**	**Year**	**Design**	**Groups**	**Primary outcome**	**Results**
Chang et al., *Surgery*. (191)	Taiwan	2020	Retrospective cohort	APA+ADX (*n* = 1,047), PA+MRA (*n* = 3,167), each subgroup matched 1:4 with EH	Stroke	ADX associated with lower risk of stroke compared to EH; MRA was not
Kim et al., *Hypertension*. (119)	Korea	2021	Retrospective cohort	PA +ADX (*n* = 755), PA +MRA (*n* = 663), each subgroup matched 1:5 with EH	NOAF	Time-dependent increases in NOAF risk in both ADX and MRA groups compared with EH.
Pan et al., *J Amer Heart Assoc*. (198)	Taiwan,China	2020	Retrospective cohort	PA +ADX (*n* = 534), PA +MRA (*n* = 1,668), matched with EH (*N* = 8,808)	AF	ADX associated with lower risk of AF compared to EH; MRA was not. ADX had a lower rate of mortality, major cardiac and cardiac/cerebrovascular events compared with MRA
Puar et al., *Clin Endocrinol (Oxf)* (199)	Singapore	2020	Retrospective cohort study	unilateral PA+ ADX (*n* = 86); unilateral PA+ MRA (*n* = 68)	A composite incident of cardiovascular events	MRA improves clinical and biochemical control, and offer similar cardiovascular protection compared with ADX
Hundemer et al., *JAMA Cardiol*. (200)	America	2018	Retrospective cohort	PA +ADX (*n* = 201), PA +MRA (*n* = 195), age-matchedEH (*n* = 40,092)	AF	PA+MRA with suppressed renin had higher risk of AF, compared to EH, PA +ADX and PA+MRA with non-suppressed renin
Hundemer et al., *lancetdiabetes endo*. (120)	America	2018	retrospective cohort	PA +MRA (*n* = 602), age-matchedEH (*n* = 41,853)	Incident of cardiovascular events	MRA had significantlyhigher risk for incident cardiovascular events and death compared with EH.
Billmann et al., *Surgery*. (201)	Germany	2020	Retrospective cohort study	Unilateral PA+pMIA (*n* = 78), unilateral PA+tMIA (*n* = 156)	Occurrence of postoperative and hypocortisolim	pMIA is comparable to tMIA in terms of clinical and biochemical, and reduced hypocortisolism and hypoglycemia.
Katabami et al., *J Hypertension*. (202)	Japan	2019	Retrospective cohort	PA with APA +ADX (*n* = 276), PA with APA +MRA (*n* = 63)	Renal function, BP, anti-hypertensive medication use	ADX associated with lower number of antihypertensive medications, higher rates of normal BP, and improved eGFR compared with MRA
Hundemer et al., *Hypertension*. (203)	America	2018	Retrospective cohort	PA +ADX (n=120), PA +MRA (*n* = 400), age and eGFR-matched EH (*n* = 15,474)	Renal function	MRA treatment is associated with annual decline in eGFR and higher risk of CKD compared to EH; ADX was not
Park et al., *Endocr J*. (204)	Korea	2017	Retrospective study	APA +ADX (*n* = 206) PA +MRA (*n* = 64)	Renalfunction and Hypokalemia	a lower postoperative eGFR and higher serum potassium levels in ADX, compared to MRA
Velema et al., *JCEM*. (190)	Dutch	2018	RCT (SPARTACUS)	PA+ADX (*n* = 92) PA +MRA (*n* = 92)	QoL	Both ADX and MRA treatment improved QoL after 1 year. ADX associated with better QoL compared to MRA treatment despite equivalent BP control

*PA, primary aldosteronism; APA, aldosterone-producing adenoma; ADX, adrenalectomy; MRA, mineralocorticoid receptor antagonist; EH, essential hypertension; AF, atrial fibrillation; NOAF, new-onset atrial fibrillation; pMIA, partial minimally invasive adrenalectomy; tMIA, total minimally invasive adrenalectomy; eGFR, estimated glomerular filtration rate; RCT, randomized clinical trial; SPARTACUS, A Randomized Trial Comparing Adrenal Vein Sampling and Computed Tomography Scan; BP, blood pressure; QoL, quality of life*.

### Cerebro-Cardiovascular Outcomes

The cardiovascular system responds dynamically to aldosterone. PA is associated with vascular and cardiac remodeling beyond the degree of BP, which is the presumed mechanism for the higher rates of cardiovascular events (3, 4, 83). A retrospective cohort study showed that patients with PA treated with MRA had a higher risk of stroke (a competing hazard ratio = 1.83, *P* < 0.001), while ADX lowered the risk of incident stroke (competing for a hazard ratio = 0.75) compared to controls with EH (191). Catena et al. reported that both ADX and treatment with MRA (spironolactone) decreased LVM in patients with PA (196). Targeted surgical and medical treatment of PA-induced LVM *via* LV inward remodeling and ADX contributed to a prominent and persistent decrease in LVMI, while medical treatment resulted in a borderline significant fall (101). Besides, the worse LV diastolic function in patients with PA could be reversed after ADX (102), and ADX exerted a beneficial effect on LV geometry and structure by reducing LV concentric geometry and the burden of LVH in patients with PA (197). In addition, ADX lowers the risks of congestive heart failure (CHF) and all-cause mortality in a long-term follow-up (112).

However, studies have shown the different effects of medical or surgical treatment on AF in patients with PA. Pan et al. demonstrated that patients with PA who underwent ADX had a lower incidence of new-onset atrial fibrillation (NOAF), but this finding was not observed in patients with PA who received a lower dose of MRA therapy (198). The authors supposed the differences between the two strategies might be caused by the different doses of MRA therapy. A higher dosage of MRA may have higher clinical efficacy and reduce the differences between ADX and MRA therapy. However, this issue needs further large prospective randomized trials to figure this out. In addition, patients with PA treated with MRA (renin remained suppressed) had a significantly higher risk of AF, where treatment of PA with MRA substantially increases renin, or with surgical ADX was associated with no significant difference in reducing the risk for developing AF (120). Accordingly, Rossi et al. also demonstrated that treatment with MRA had a higher incidence of AF, compared with both ADX treatment for PA and the EH group (192), which was confirmed by a recent meta-analysis. This meta-analysis enrolled 2,705 patients with PA, and the results demonstrated that the incidence of NOAF among the patients with PA receiving treatment with MRA was much higher compared to those inpatients for PA who underwent ADX (pooled OR: 2.83, 95% CI: 1.76–4.57 in the random-effects model). The pooled OR was 1.91 (95% CI: 1.11–3.28) when compared to the patients with EH (205). These data suggested that persistent renin suppression may serve as a biomarker for inadequate MR blockade, leading to ongoing aldosterone exposure and consequent cardiometabolic effects. Taken together, surgical treatment seems to have a better protective effect on AF than medical treatment in patients with PA.

### Renal Outcomes

The renal system is a direct target organ of hyper aldosteronism, and patients with PA often have an association with decreased eGFR and CKD (139, 142, 144). Effective treatment with either surgery or MRA therapy will unmask the underlying CKD in patients with PA (206). Short- and long-term follow-up after ADX consistently indicated that early involvement of the kidney in patients with PA was characterized by functional changes that would be largely reversible, with a significant decrease in urinary albumin excretion (207), and microalbuminuria was more likely to subside to normal levels (204). Besides, ADX had significantly lower utilization of antihypertensive medications, higher rates of normalization of BP, and improved eGFR compared with treatment with MRA (202). However, for elderly patients, MRA therapy may be more appropriate (204). Of course, a few major cardiovascular events and mortality events were also observed after surgical treatment (208). Studies found that kidney function further deteriorated after ADX in patients with PA (209, 210), and the incidence and risk of postoperative acute kidney injury were significantly higher in patients with PA after surgical ADX (211). MRAs must be dosed with care in the setting of CKD, as they may precipitate hyperkalemia [49]. Therefore, clinicians should pay more attention to postoperative renal function in patients with PA at elevated risk for a decline in kidney function, and it is important to evaluate the age, preoperative PAC, and preoperative potassium level before choosing the method of treatment.

### Other Outcomes of the Vascular System

Arterial stiffness is one of the important factors in patients with PA and severe arterial stiffness before surgery was significantly associated with renal function decline and less LVM regression after ADX in patients with lateralized PA (212). Strauch et al. found that the PWV significantly decreased after ADX, whereas there are no changes in arterial stiffness (PWV, augmentation index) indices in patients treated with MRA (spironolactone) (213). Another study also showed both brachial-ankle PWV (baPWV) and heart-ankle PWV (haPWV) were significantly reduced after ADX in patients with APA, which suggested that ADX could reverse adverse vascular changes in patients with APA (127).

### Major Limitations and Disadvantages of Treatments

The treatment objectives for patients with PA include resolution of hypokalemia and prevention of the morbidity and mortality associated with hypertension, progressive CKD, and further TOD (cerebro-cardiovascular, renal, and vascular systems). Although the surgery would improve the QoL of patients to normal population-adjusted values, it is more cost-effective in the long term, because the patients require fewer medications and less frequent clinic visits (190). Besides, surgery may also bring about superior cardiovascular, renal, and metabolic outcomes (214, 215). However, there exist some limitations and disadvantages to the surgical treatment (i.e., ADX) of PA. Firstly, not all patients with PA are suitable for ADX, and ADX is the best choice for common unilateral APA (aldosteronoma), primary unilateral adrenal hyperplasia, and multinodular unilateral adrenocortical hyperplasia. Secondly, there exists a risk of short-term hypoaldosteronism, leading to clinically important hyperkalemia after ADX surgery (216, 217). Thirdly, because glomerular hyperfiltration associated with aldosterone excess might mask mild-to-moderate underlying renal failure, most patients with long-standing PA have some degree of renal insufficiency. Some studies demonstrated that kidney function further deteriorated after ADX (209, 210) and the incidence and risk of postoperative acute kidney injury were significantly higher after ADX in patients with PA (211). For medical treatment, it is essential that the MRA dosage must be adequate to fully block the toxic effects of hyperaldosteronism. Nevertheless, for unselective MRA (spironolactone, canrenone, potassium canrenoate), painful gynaecomastia, erectile dysfunction, and decreased libido in men are induced in PA patients with spironolactone at dosages of more than 50 mg/day, because spironolactone and its metabolites are antagonists of the androgen receptor. They also are agonists of the progesterone receptor and thus interfere with the estrus cycle. These off-target effects occur at 25 mg/day after chronic use in some patients. Besides, agonist activity at the progesterone receptor may result in menstrual irregularity in women (218). Eplerenone, a competitive and selective MRA, is more expensive, weaker, and shorter-acting than the other MRAs. Most importantly, in patients with PA, lifetime use of MRA could increase the pill burden of antihypertensive medications significantly and reduce QoL greatly.

## Conclusions and Perspectives

Primary aldosteronism (PA) is the most common cause of endocrine hypertension, and hyperaldosteronemia is one of the important characteristics for patients with PA (219). Increasing experimental and clinical data suggest prolonged exposure to elevated aldosterone concentrations in patients with PA is associated with increased TOD, including brain, heart, kidney, vessels, adipose tissues, and OSA, and therefore, leads to an increase in morbidity from cerebro-cardiovascular events. To attenuate the toxic effects of excess aldosterone, the first and key step is to screen for PA, especially in patients with hypertension that is severe and/or resistant to treatment. Early diagnosis is the fundamental and critical point to obtain benefits from the prevention of the development of TOD, because ADX for patients with unilateral could bring BP under control with the withdrawal or prominent reduction in the number and dosage of antihypertensive medications, as well as even reverse TOD (220). Besides, titration of MRA therapy to increase plasma renin levels may be an effective approach to avoid excessive cardiovascular risk in medically treated patients with PA. Furthermore, there are novel third-generation nonsteroidal MRAs (like eplerenone, the receptor selectivity of AZD-9977, esaxerenone, finerenone, and KBP-5074) currently in clinical trials and provide a cardiorenal benefit above that of current optimized standard-of-care treatment in a high-risk population with reduced renal function, and with a lower risk of hyperkalemia (221–224). These nonsteroidal MRAs generally exhibit both high affinity and good selectivity for the MR over other steroid receptors may have a better safety profile in patients with PA (222–226). For example, finerenone from Bayer is non-steroidal, and suggested to be cardiac preferring (over the kidney) in contrast with the steroidal MRAs (227). Esaxerenone from Daiichi-Sankyo showed over 10 times as potent as eplerenone in terms of lowering blood pressure: its particular clinical significance claims to be a safe and effective antihypertensive in patients with moderate renal dysfunction and/or T2DM and albuminuria (228). Therefore, these novel nonsteroidal MRAs have better therapeutic options for patients with PA in the future (222–224). In addition, more accurate methods to select patients with unilateral PA for ADX are needed in medically treated patients in the future (183). Taken together, there should be a low threshold for initiating screening for PA, since the early diagnosis of PA and early initiation of specific treatment strategies have a substantial impact on reducing cerebro-cardiovascular events and mortality in the long term.

## Author Contributions

JL and L-QY conceived and designed the manuscript. XL collected the literature and wrote the manuscript. MU, XW, FX, S-KS, and L-ML were involved in data collection and made substantial contributions to data analysis. All the authors read and approved the manuscript and agreed to its publication.

## Funding

This work was supported by grants from the National Natural Science Foundation of China (Nos. 82100944, 82100494, 82070910, and 81770881), Natural Science Foundation of Hunan Province (No. 2021JJ40842), and the Clinical Research Center for Medical Imaging in Hunan Province of China (No. 2020SK4001).

## Conflict of Interest

The authors declare that the research was conducted in the absence of any commercial or financial relationships that could be construed as a potential conflict of interest.

## Publisher's Note

All claims expressed in this article are solely those of the authors and do not necessarily represent those of their affiliated organizations, or those of the publisher, the editors and the reviewers. Any product that may be evaluated in this article, or claim that may be made by its manufacturer, is not guaranteed or endorsed by the publisher.
